# Physiological and Pathophysiological Roles of IgM Fc Receptor (FcµR) Isoforms

**DOI:** 10.3390/ijms24065728

**Published:** 2023-03-17

**Authors:** Hiromi Kubagawa, Caren Clark, Christopher M. Skopnik, Pedram Mahmoudi Aliabadi, Khlowd Al-Qaisi, Ruth Teuber, Peter K. Jani, Andreas Radbruch, Fritz Melchers, Niklas Engels, Jürgen Wienands

**Affiliations:** 1 Deutsches Rheuma-Forschungszentrum, 10117 Berlin, Germany; 2 Institute of Cellular & Molecular Immunology, University Medical Center, 37073 Göttingen, Germany

**Keywords:** Ig-tail tyrosine (ITT) motif, single-cell RNA sequence (scRNAseq), chronic lymphocytic leukemia (CLL), autoimmune diseases, soluble FcµR

## Abstract

IgM is the first antibody to emerge during phylogeny, ontogeny, and immune responses and serves as a first line of defense. Effector proteins interacting with the Fc portion of IgM, such as complement and its receptors, have been extensively studied for their functions. IgM Fc receptor (FcµR), identified in 2009, is the newest member of the FcR family and is intriguingly expressed by lymphocytes only, suggesting the existence of distinct functions as compared to the FcRs for switched Ig isotypes, which are expressed by various immune and non-hematopoietic cells as central mediators of antibody-triggered responses by coupling the adaptive and innate immune responses. Results from FcµR-deficient mice suggest a regulatory function of FcµR in B cell tolerance, as evidenced by their propensity to produce autoantibodies of both IgM and IgG isotypes. In this article, we discuss conflicting views about the cellular distribution and potential functions of FcµR. The signaling function of the Ig-tail tyrosine-like motif in the FcµR cytoplasmic domain is now formally shown by substitutional experiments with the IgG2 B cell receptor. The potential adaptor protein associating with FcµR and the potential cleavage of its C-terminal cytoplasmic tail after IgM binding are still enigmatic. Critical amino acid residues in the Ig-like domain of FcµR for interacting with the IgM Cµ4 domain and the mode of interaction are now defined by crystallographic and cryo-electron microscopic analyses. Some discrepancies on these interactions are discussed. Finally, elevated levels of a soluble FcµR isoform in serum samples are described as the consequence of persistent B cell receptor stimulation, as seen in chronic lymphocytic leukemia and probably in antibody-mediated autoimmune disorders.

## 1. Introduction

Two separate lineages of lymphocytes are generated in their distinctive tissue sites for adaptive immunity. B cells are developed in the bone marrow in mammals and the bursa of Fabricius in chickens and contribute to humoral immunity, whereas T cells are generated within the thymus and contribute to cellular immunity [1]. Antibody or immunoglobulin (Ig) is a key player in the humoral immunity and has a dual-binding activity: usually first to antigens via its two identical N-terminal variable domains in the Fab region and then to effector molecules via its constant region in the Fc region (except for IgE–FcεRI interaction). Of five different antibody classes, IgM is the first antibody class to appear during phylogeny (from jawed vertebrate onward), ontogeny (from the 8th–9th week of gestation in humans), and in immune responses (a few days after antigen exposure). On the surface of B cells, monomeric IgM is part of the B cell antigen receptor (BCR) [2], and at their terminally differentiated plasma cells, secreted pentameric IgM initiates for humoral immunity. It is evident from studies of mice unable to secrete IgM that both pre-immune “natural” and antigen-induced “immune” IgM are important for protective immunity and regulation of autoimmune processes by recognizing pathogens and self-antigens [3,4]. Effector molecules interacting with the Fc region of IgM, such as complement and its receptors, have thus far been extensively studied for IgM-mediated protection and immune regulation [5,6]. In contrast, studies investigating the role of the IgM Fc receptor (FcµR) in IgM effector functions have been limited, which is in part due to its identification only ~14 years ago [7]. Since many review articles on FcµR have already been published elsewhere [4,8,9,10,11,12,13], this article briefly reiterates the unique features and unresolved issues of FcµR and discusses contradicting views on various aspects of this receptor.

## 2. Disputes on the Cellular Distribution of FcµR

### 2.1. Questioning Whether FcµR Is Expressed by Mouse Innate Immune Cells

Most of the available evidence indicates that unlike FcRs for switched Ig isotypes, which are expressed by a variety of immune and non-hematopoietic cell types [13,14], FcµR is more selectively expressed by B cells, T cells, and, to a lesser extent, NK cells in humans, and only B cells in mice. This conclusion is based on various analyses: (i) flow cytometric assessments using IgM ligands and FcµR-specific monoclonal antibodies (mAbs) to stain both freshly prepared primary cells and established cell lines before and after activation with various stimuli ex vivo, (ii) biochemical characterization of proteins isolated from cellular lysates using both IgM ligands and mAbs, and (iii) sensitive reverse transcription polymerase chain reaction (RT-PCR) assays detecting FcµR transcripts [7,9,10,13,15]. However, a considerable number of papers have also described the expression of FcµR by non-B cells in mice (i.e., granulocytes, monocyte/macrophages, dendritic cells (DCs), and Th17 cells), mainly based on the functional alterations in these cell types in a particular strain of the *Fcmr*-deficient (KO) mouse line (designated as *Fcmr* KO-M in this article) [16,17,18]. 

**Figure 1:**
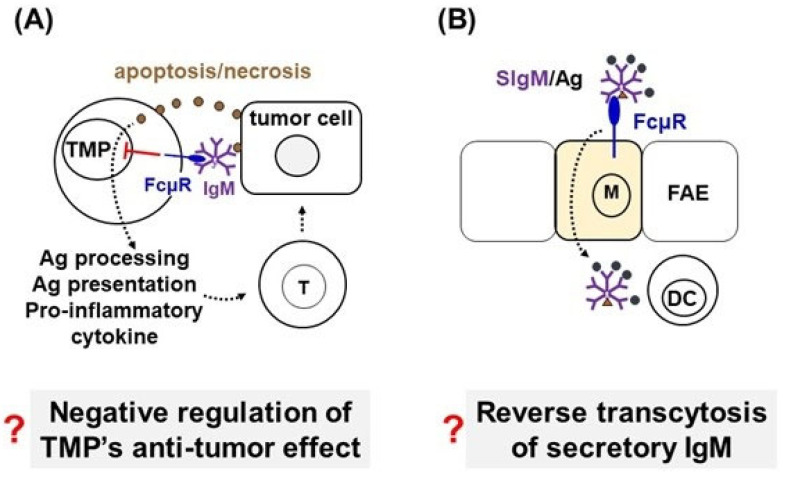
Disputes on the cellular distribution of FcµR in mice. (**A**) Questioning the negative regulation of tumor growth by FcµR expressed on tumor-associated mononuclear phagocytes (TMPs). TMPs engulf cell debris from apoptosis or necrosis of tumor cells and process and present antigens to T cells and produce proinflammatory cytokines. FcµR (blue tennis racket) expressed by TMPs inhibits this process by interacting with IgM anti-tumor antibody (purple pentamer). (**B**) Questioning the reverse transcytosis of secretory IgM (SIgM)/antigen complexes via FcµR expressed by the apical portion of microfold (M) cells in the follicle-associated epithelium (FAE) from the intestinal lumen to dendritic cells (DC). SIgM is shown as a purple pentamer plus a triangle secretory component and antigens as black circles.

Recently, Kubli et al. described that FcµR negatively regulated anti-tumor activity by tumor-associated mononuclear phagocytes (TMPs) in a melanoma mouse model using the *Fcmr* KO-M strain. The mutant mice had increased numbers of TMPs, a reduced tumor size, and showed improved survival as compared with control wildtype (WT) mice. Single-cell RNA sequence (scRNAseq) analysis of TMPs from *Fcmr* KO-M and WT mice revealed a unique TMP subset with enhanced antigen processing/presenting properties in the mutant mice [19]. However, when we reexamined the scRNAseq data of TMPs, we found no *Fcmr* expression by such TMPs [20]. Almost none of the analyzed TMPs (8000 *Fcmr* KO-M and 6352 *Fcmr* WT cells) had *Fcmr* transcript reads. Notably, one WT cell with four *Fcmr* transcript reads was found to also contain transcript detection of B cell-specific genes. These results are consistent with the findings from the comprehensive scRNAseq analyses of various mononuclear phagocytic populations conducted by Hume and his colleagues, showing that *Fcmr* transcripts were a marker for a B cell-specific contaminant cluster [21,22]. In reply to our aforementioned comments [20], Kubli et al. assumed a technical limitation as a reason for the lack of *Fcmr* transcripts in TMPs, a so-called dropout effect, and emphasized on the surface-staining data of FcµR expressed on myeloid cell as determined by a ‘novel’ rat antiserum, instead of mAb, which was raised against a recombinant soluble FcµR protein [23]. Unfortunately, there was no demonstration of the specificity of such polyclonal antiserum by immunoprecipitation and proteomic analyses. Thus, there is no solid evidence for the expression of FcµR by myeloid cells, including TMPs (see Figure 1A). It is also noteworthy that many kinds of artifacts may occur in scRNAseq analyses, including the fragmentation of tissue-resident macrophages during isolation, contamination, stochastic sampling, and even non-zero sequencing of DNA fragments [21,22]. Retrospectively, it seems likely that the functional alterations observed in non-B cell populations of this particular *Fcmr* KO-M mouse resulted from an off-target effect or the consequence of the *Fcmr* ablation procedures unrelated to the inactivated *Fcmr* itself (see below). 

### 2.2. Reverse Transcytosis of Secretory IgM via FcµR Expressed on M Cells in Peyer’s Patches

Microfold (M) cells reside in the follicle-associated epithelium at Peyer’s patches and have a unique function to deliver a variety of materials, including particulate antigens, soluble macromolecules, and pathogens from the intestinal lumen to Peyer’s patches [24,25]. In addition to sampling of these heterogenous materials by M cells, immune complexes of secretory IgA (SIgA) and antigens in intestinal lumens have been shown to be reversely transcytosed through the mucosal epithelium by selective binding to receptors, Dectin-1 (C-type lectin) and Siglec-5 (sialic acid binding Ig-like lectin 5), expressed on the apical surface of M cells [26]. Recently, the same group of authors extended this receptor-mediated, reverse transcytosis to secretory IgM (SIgM) and found that FcµR was expressed on the apical surface of M cells and was involved in the M cell-mediated reverse transcytosis of SIgM/antigen complexes, suggesting a regulatory role of FcµR in mucosal immunity [27] (see Figure 1B). However, IgM can bind various proteins other than FcµR (e.g., CD22/siglec-2, tripartite motif-containing protein 21 (TRIM21)/E3 ubiquitin ligase, apoptosis inhibitor of macrophages (AIM)/soluble protein α/CD5L, Fcα/µR, polymeric Ig receptor (pIgR), mannan binding protein, binding Ig protein (Bip)/heat shock protein A5 (HSPA5)/78 kDa glucose-regulated protein (GRP78)). Moreover, preliminary findings in collaboration with Takashi Kanaya and Hiroshi Ohno (RIKEN, Yokohama, Japan; unpublished) have raised the question of whether FcµR is indeed expressed by M cells in follicle-associated intestinal epithelium. Thus, the validity of the above findings of M cell-mediated reverse transcytosis of SIgM via FcµR must await further confirmation.

## 3. Common and Conflicting Findings in *Fcmr*-Deficient Mice

**Figure 2:**
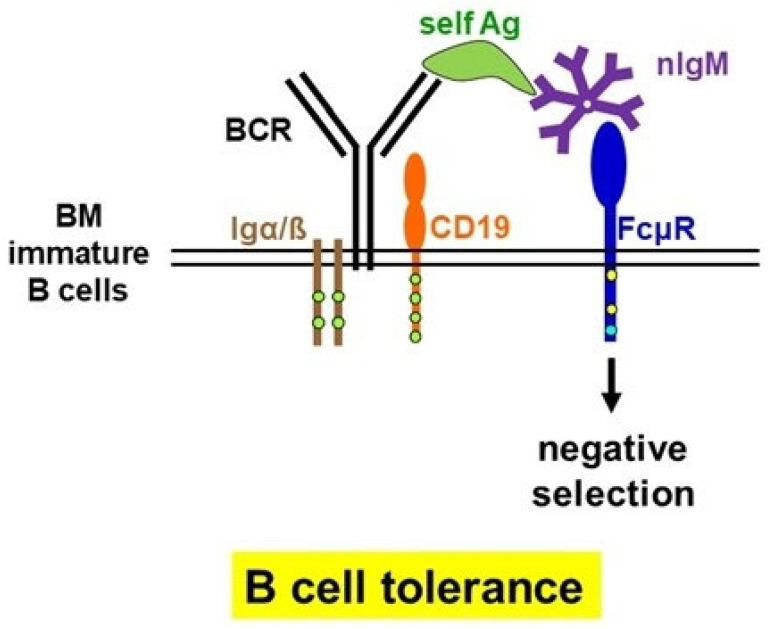
Regulatory role of FcµR in B cell tolerance. The common finding in all five strains of FcµR-deficient mice is an impairment of B cell tolerance. As a proposed model, the *cis* engagement of IgM B cell receptor (BCR) and FcμR via immune complexes of self-antigen (green) with natural pentameric IgM (purple) on immature B cells in the bone marrow contributes to the negative selection. Igα/Igß (purple lines) with ITAM (green circles) and CD19 (orange) are also shown. The details are described elsewhere [30].

So far, five different strains of *Fcmr* KO mice have been developed by four different laboratories using different targeting strategies, and at least eight different groups of investigators have examined their resultant phenotypes, with some discrepancies (see reviews [12,13]). However, the common finding among these mutant mice is an impairment of B cell tolerance, as evidenced by enhanced serum titers of autoantibodies of both IgM and IgG [12,13] (Figure 2). The major discrepancy among the reported phenotypes is mainly attributed to the functional alterations of non-B cell populations observed in the *Fcmr* KO-M strain [13]. The *Fcmr* KO-M mice are unique among all *Fcmr* KO strains in their extensive deletion of genomic DNA (~10 kb), ranging from exon 2 (signal peptide-2 and Ig-like domain) to exon 8 (cytoplasmic tail-3 and 3′ untranslated region). Moreover, the *Neo* resistance cassette remained in the mouse genome [16], a situation that is known to impact on mouse phenotypes. According to the epigenetic analysis of the *Fcmr-Il10* locus in regulatory T (Treg) cells by Ohkura and Sakaguchi [28], three loci, the 3′ region of *Fcmr* (exon 5 (transmembrane) to exon 8), and 5′ upstream of *Il10* and *Il10*, were selectively in an open chromatin configuration when Treg cells were activated; hence, such loci might be highly accessible to transcription factors [13]. If this selective epigenetic alteration seen in Treg cells is also the case for myeloid cells including TMPs, it may account for different myeloid functions in the *Fcmr* KO-M strain (missing this 3′ region of *Fcmr*) as compared with its WT control and other *Fcmr* KO strains. Supporting this, a difference in granulocyte function was noted between *Fcmr* KO-M and our KO (which lack exons 2–4 [29]) mice. Production of reactive oxygen species was significantly higher in the former KO granulocytes than WT controls upon stimulation with N-formylmethionine-leucyl-phenylalanine in the presence or absence of lipopolysaccharide (see Figure 3C in [16]), but was comparable in our *Fcmr* KO and WT granulocytes [20]. Collectively, any alterations in non-B cell functions observed in the *Fcmr* KO-M strain [16,17,18] most likely are not due to the lack of FcµR per se but to an off-target effect caused by the extensive targeting strategy as compared to that of other *Fcmr* mutant strains.

## 4. Structural and Signaling Aspects of FcµR

### 4.1. Dual-Signaling Potential of FcµR

**Figure 3:**
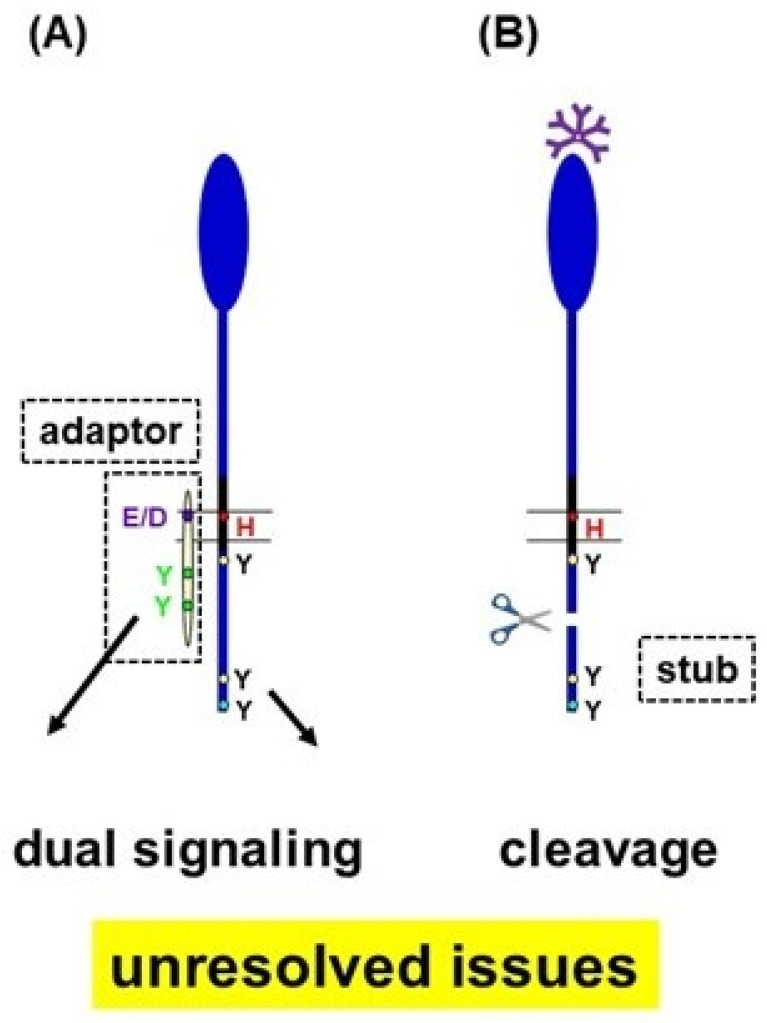
Unresolved issues of FcµR. (**A**) Existence of a potential adaptor protein non-covalently associating with FcµR via the His**^253^** in its TM. (**B**) Potential cleavage at the C-terminal tail of FcµR, after receptor ligation, creating the stub containing the ITT motif.

FcµR is encoded by a single copy gene located on chromosome 1q32.2 adjacent to those encoding two promiscuous IgM-binding receptors, pIgR and Fcα/µR. FcµR is a type I transmembrane sialoglycoprotein with an *M*_r_ of ~60 kDa, which consists of a V-set Ig-like domain responsible for exclusive Fcµ binding, an additional extracellular (EC) stalk region, a transmembrane (TM) segment containing a charged His residue at position 253 (H**^253^**), and a relatively long cytoplasmic portion containing evolutionary conserved, three Tyr, and five Ser residues [7]. Among these Tyr residues, the C-terminal Y**^385^** matches the Ig-tail Tyr (ITT) motif (DYxN, where x indicates any amino acid (aa)) seen in the cytoplasmic tails of membrane-bound IgG and IgE [31,32]. The two C-terminal Tyr residues Y**^366^** and Y**^385^** of FcµR were found to be involved in FcµR-mediated endocytosis [33,34], whereas the membrane proximal Y**^315^** was involved in the receptor-mediated protection from agonistic IgM anti-Fas mAb-induced apoptosis in vitro [34]. When the fate of IgM bound to FcµR on stable transductants expressing WT or H253F (a point mutation of His at position 253 to Phe) mutant form was examined by immunofluorescence microscopy, the mutant showed enhanced cap formation even at 4 °C, suggesting an anchoring role of the H**^253^** residue of FcµR in the plasma membrane [34]. Since we have so far successfully developed FcµR (membrane form) stable transductants using various lymphoid cell lines as hosts, we initially considered that, unlike activating isoforms of paired receptors, FcµR could be displayed on the plasma membrane without an adaptor protein that carries a negatively charged aa residue in its TM to neutralize the H**^253^** residue of FcµR. Alternatively, the relatively long cytoplasmic part of FcµR might compensate for such adaptor-dependent surface expression. However, when murine 3T3 fibroblasts were transduced with a retroviral bicistronic expression vector encoding FcµR and green fluorescein protein (GFP) cDNAs, the resultant GFP**^+^** cells did not express FcµR on their cell surface, suggesting the requirement of companion molecules, not present in fibroblasts, for FcµR to reach the plasma membrane (Marie Burns and HK, unpublished). This scenario is reminiscent of our findings with paired Ig-like receptors (PIRs) that the FcR common γ chain is a prerequisite for the cell surface expression of PIR-A (short cytoplasmic tail, activating isoform), but not of PIR-B (long cytoplasmic tail, inhibitory isoform), in fibroblasts [35]. Whether or not such a companion molecule (e.g., an adaptor protein non-covalently associating with FcµR) exists remains to be elucidated (Figure 3A). 

Another unresolved issue is the enhanced migration of phosphorylated FcµR on sodium dodecyl-sulfate polyacrylamide gel electrophoresis (SDS-PAGE) upon receptor ligation or treatment of FcµR-expressing cells with the tyrosine phosphatase inhibitor pervanadate [7]. Pronounced phosphorylation of FcµR at both Tyr and Ser residues might cause a global structural change, leading to faster migration on SDS-PAGE, unlike the usually observed slower migration. Alternatively, it might be due to proteolytic cleavage of FcµR, after receptor ligation. Preliminary findings with epitope-tagged FcµR transductants suggested a potential cleavage of its cytoplasmic tail (Eugene J. Becker, Yoshiki Kubagawa, Marie Burns, and HK, unpublished). In this regard, ligation of the FcγRIIa, an activating FcγR, on platelets leads to activation of both the metalloprotease that targets the collagen receptor glycoprotein VI to shed its ectodomain and the intracellular calpain that cleaves the cytoplasmic tail of FcγRIIa to remove the ITAM-containing stub. This finding suggests a potentially novel mechanism for platelet dysfunction by FcγRIIa after immunological insult, including IgG autoantibodies to platelets [36]. The precise molecular basis for the enhanced migration of phosphorylated FcµR awaits further investigation (Figure 3B). 

Collectively, unlike pairs of activating and inhibitory receptors, FcµR itself may possess a dual-signaling ability. One results from a putative ITAM-bearing adaptor protein, which may non-covalently associate with FcµR via H**^253^**, and another one involves Tyr and/or Ser residues within the cytoplasmic FcµR domain. 

### 4.2. Signaling Function of the Ig-Tail Tyrosine (ITT)-like Motif of FcµR

**Figure 4:**
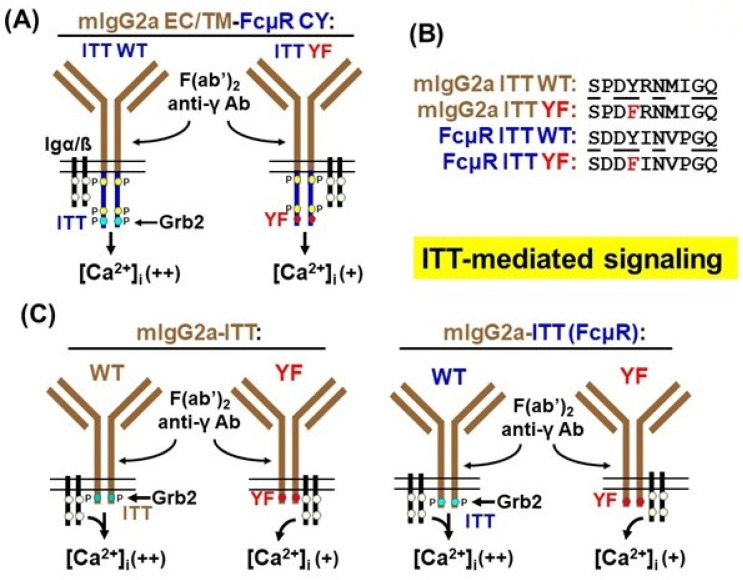
Signaling function of the ITT-like motif of FcµR. (**A**) Chimeric IgG2a BCR consisting of the extracellular and transmembrane regions of membrane IgG2a (mIgG2a; brown) and the cytoplasmic tail of FcµR (blue), along with Igα/ß (black). ITT tyrosines (light blues) and other tyrosines (light yellow circles). Point mutation of the ITT tyrosine to phenylalanine (YF; red circles). Intracellular Ca^2+^ concentration ([Ca^2+^]_i_) and recruitment of Grb2 after cross-linkage of the chimeric mIgG2a BCR by F(ab’)_2_ fragments of anti-γ antibodies are indicated. Potential phosphorylation (P) of chimeric IgG2a BCR are also shown based on protein blot analyses. (**B**) Amino acid sequence alignments around the ITT motif of mIgG2a of WT (top row) or YF variants (2nd row) as compared with the ITT motif of FcµR WT (3rd row) or YF (4th row). (**C**) Signaling function of mIgG2a-ITT of WT (1st column) or YF (2nd column), and of mIgG2a-ITT (FcµR) of WT (3rd column) or YF (4th column).

To determine if the ITT-like motif (DY**^385^**INV) in FcµR indeed has the capacity to transduce intracellular signaling, we tested if the cytoplasmic tail of FcµR can functionally compensate for that of membrane-bound IgG (mIgG). To this end, we replaced the cytoplasmic domain of mIgG2a with that of FcµR and expressed the resulting chimeric BCR in the IgM-bearing human B cell line DG75 (mIgG2a/FcµR WT). As a control, we inactivated the FcµR ITT-like motif by replacing the central Tyr at position 385 with phenylalanine (mIgG2a/FcµR Y385F) (hereafter, mIgG2a/FcµR WT and mIgG2a/FcµR Y385F are designated as WT and YF, respectively, for simplicity). Both WT and YF stable transductants expressed similar levels of mIgG2a-BCRs on their cell surfaces. To test the signaling capacities of the chimeric mIgG-BCRs, we analyzed mobilization of the second messenger Ca^2+^ on BCR activation. Ligation of the chimeric mIgG2a-BCRs induced significantly stronger Ca^2+^ mobilization in the WT than in the YF mutant transductants, suggesting a signal-amplifying function of the ITT-like motif of FcµR, similar to that of wildtype mIgG-BCRs (Figure 4A). Importantly, the different Ca^2+^ mobilization kinetics were not due to cell-intrinsic variations since stimulation of endogenous mIgM-BCRs gave rise to similar Ca^2+^ mobilization profiles in both transductants, of which the surface IgM levels were indistinguishable. Western blot analyses revealed that chimeric mIgG2a/FcµR protein of 62 kDa was tyrosine-phosphorylated post-ligation in both WT and YF transductants. These results indicate that while the ITT-like motif of FcµR can functionally replace the ITT of mIgG2a-BCRs, there are additional tyrosine residues in the FcµR cytoplasmic domain that undergo phosphorylation when brought into the immediate proximity of an activated BCR. However, only the ITT-like motif seems to interact with the adaptor protein Grb2, since it was co-precipitated with ITT-WT but not -YF mutant mIgG2a/FcµR fusion proteins.

The functional core of ITT-like motifs consists of the aa sequence YxN [32]. To investigate the signaling impact of aa that flank the FcµR ITT-like motif in more detail, we replaced the aa surrounding the ITT core motif of mIgG2a with those of the FcµR (Figure 4B). The resulting mIgG2a-ITT (FcµR) chimeric variants contained the modified ITT sequence SDD**Y**INVPGQ or the phosphorylation-deficient sequence SDD**F**INVPGQ (hereafter referred to as mIgG2a-ITT (FcµR) WT or YF, respectively). These constructs (Figure 4C, right), as well as mIgG2a ITT WT or YF mutants as controls (Figure 4C, left), were expressed and analyzed in DG75 B cells, as above. The resultant transductants expressed very similar levels of mIgG2a-BCRs on their cell surfaces. In both cases, stimulation of the WT versions of mIgG2a-BCRs gave rise to much stronger Ca^2+^ mobilization profiles than in cells expressing the YF mutant mIgG2a-BCR variants (Figure 4C). As before, stimulation of endogenous mIgM-BCRs confirmed that these differences were not due to cell-intrinsic variations. Notably, anti-phosphotyrosine Western blot analysis of purified mIgG2a-BCRs showed a much stronger ITT phospho-signal, along with enhanced co-precipitation of Grb2 in cells expressing the modified mIgG2a-ITT (FcµR) BCR as compared to cells expressing the WT mIgG2a-ITT BCR. These results indicate a more efficient phosphorylation of the FcµR ITT-like motif than that of the mIgG2a ITT, which goes along with its more potent Grb2 recruiting activity. This improved ITT phosphorylation may be caused by the additional Asp residue in the minus 2 position relative to the Tyr. However, it does not seem to further enhance amplification of Ca^2+^ mobilization.

Collectively, these findings clearly demonstrate that the ITT-like motif of FcµR has the ability to mimic the signaling function of the mIgG2a ITT. The amino acids surrounding the FcµR ITT appear to make the central tyrosine residue an even more favorable substrate for B cell-expressed protein tyrosine kinases such as Src and/or Syk, resulting in a more potent recruitment of Grb2. Furthermore, besides the ITT-like motif, there are two additional evolutionary conserved tyrosine residues in the cytoplasmic domain of FcµR, one of which, Y**^366^**, is embedded in a sequence (E**Y**VSL) that resembles known phosphorylation and SH2 domain binding sites. Our results show that tyrosine residues other than that in the ITT-like motif are phosphorylated when the FcµR cytoplasmic domain is brought into proximity of activated BCRs. This raises the possibility that, for example, Y**^366^** serves as a phospho-acceptor and SH2 domain docking site to regulate additional intracellular signaling pathways. It remains to be elucidated how the Y**^385^** ITT-mediated signals and the aforementioned potential cleavage in the cytoplasmic tail of FcµR after ligation work. 

### 4.3. Identification of Amino Acid Residues of FcµR Critical for IgM Binding

Using transductants stably expressing human or mouse FcµR, we observed different properties of the two orthologues in constitutively or transiently binding their IgM ligands, respectively [9]. Via domain swapping analysis, this difference was directly attributed to the ligand-binding Ig-like domain rather than other parts of FcµR. The aa sequences of the Ig-like domain of human and mouse FcµRs were thus aligned with each other based on the secondary structure of human pIgR domain 1 (D1), determined by crystallography (PDB 5D4K) [37,38]. Several sequence differences were found around the putative ligand-binding complementary determining regions (CDRs) that could account for the IgM binding difference between human and mouse receptors. In addition, two distinct stretches of 4-aa and 5-aa residues were also found in the presumed A ß strand and DE loop of FcµR, respectively [39]. Based on this sequence comparison, we hypothesized that if non-conserved aa residues in human FcµR are replaced by the corresponding mouse residues, then the resultant human FcµR mutants may no longer constitutively bind IgM, similar to mouse FcµR. We thus replaced non-conserved aa residues of human FcµR with mouse equivalents and generated transductants stably expressing mutant or WT receptors. The eight different resultant FcµR mutant transductants were compared with WT transductants for their cell surface expression and IgM binding by flow cytometry using receptor-specific mAbs and IgM paraproteins as ligands. Since the details about receptor levels and IgM binding activity have been described elsewhere [39], the main findings are summarized below.

**Figure 5:**
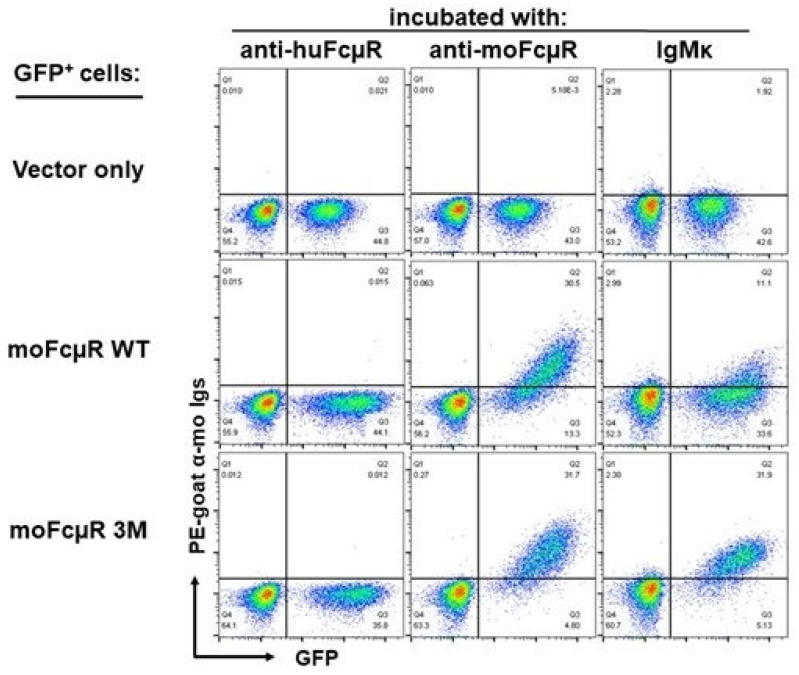
Flow cytometric analysis of FcµR expression and IgM binding by transductants. BW5147 thymoma cell lines were transduced by retroviral bicistronic expression constructs containing only GFP cDNA (vector only; top row) or GFP cDNA and mouse FcµR cDNA of wildtype (WT; middle row) or three site-directed mutations at -66N, TPCLD78-82KQYPR, and K108N (3M; bottom row), before enriching GFP**^+^** cells by FACS and establishing stable transductants. An equal mixture of GFP-negative control BW5147 cells and GFP-positive transductants was incubated with IgG1κ mAbs specific for human FcµR (as an isotype-matched control; left column) or mouse FcµR (middle column), or with IgM ligand (right column). Bound mAbs or IgM were developed by addition of polyvalent PE-labeled goat anti-mouse Ig antibodies. A representative flow cytometric profile is depicted from five different experiments. Note the significant difference (*p* < 0.05) in the mean fluorescence intensities of IgM binding between moFcµR WT and 3M transductants, as determined by Student’s *t* test.

Three sites of human FcµR, i.e., Asn**^66^** in the presumed CDR2, Lys**^79^**-Arg**^83^** in the DE loop, and Asn**^109^** in the CDR3, were responsible for its constitutive IgM binding since diminished IgM binding was observed for each replacement mutant without alteration of receptor expression levels. Notably, as previously presented [40], when all the above three sites of human residues were used to replace the mouse equivalents, the resultant mouse FcµR triple mutant (-66N, TPCLD78-82KQYPR, K108N) was found to constitutively bind IgM, such as human FcµR (Figure 5). The latter finding strongly supported that the aforementioned three sites were indeed critical for constitutive binding of IgM to human FcµR. Intriguingly, substitutions of Glu**^41^** and Met**^42^** in the CDR1, which is shorter than that of IgM- and IgA-binding receptors (pIgR and Fcα/µR), with the corresponding murine aa residues Gln and Leu, either alone or more prominently in combination, enhanced both the receptor levels and IgM binding activity. This finding was unexpected, because the mouse FcµR had limited or transient IgM binding activity as compared with human FcµR. While the molecular basis for this enhancement remains to be elucidated, this serendipitous finding may turn out to be informative when considering the design of therapeutic interventions targeting FcµR. A four-aa stretch of Lys**^24^**-Gly**^27^** in the predicted A ß strand of human FcµR appeared to be essential for maintenance of its receptor conformation on plasma membranes because of the reduction of both receptor expression and IgM binding potential when these were mutated. 

Collectively, the results from the site-directed mutational analyses suggest that the Asn residues at both positions 66 and 109 in the CDR2 and CDR3, respectively, and the stretch from Lys to Arg at positions of 79 to 83 in the DE loop of human FcµR, are responsible for its constitutive IgM-ligand binding activity.

### 4.4. Crystallographic Analysis of Human FcµR

**Figure 6:**
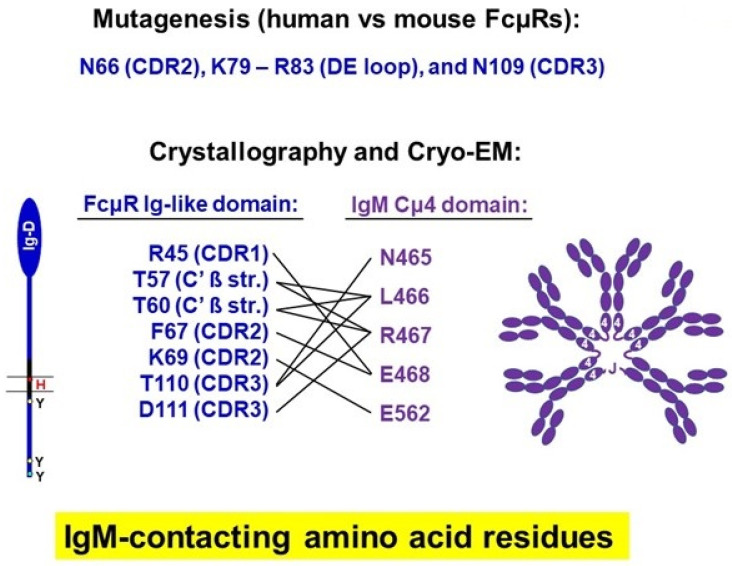
Identification of amino acid residues in the interaction of FcµR and IgM. Top: The three indicated sites are determined by site-directed mutagenesis based on the IgM-binding difference between human and mouse FcµRs. Bottom: Seven different amino acid residues (single-letter codes; blue) in the Ig-like domain of FcµR are listed as critical residues for binding to the amino acid residues (purple) of the IgM Cµ4 domain, as determined by crystallographic and cryo-EM analyses. Complementary determining region (CDR) and ß strand (ß str.).

Recently, two groups of investigators, Junyu Xiao et al. (Peking University, Beijing) and Peter Rosenthal et al. (Francis Crick Institute, London), have independently released their pre-reviewed results of the structural basis for the interaction of FcµR with IgM, as determined by crystallography and cryo-electron microscopy (cryo-EM). The data are publicly available at the URLs provided at the end of the text. Although there were some differences in results (especially regarding stoichiometry) between these two studies, the essential findings were as follows. FcµR predominantly bound to the fourth constant domain (Cµ4) of IgM molecules. The key IgM-contacting aa residues of FcµR were: Arg**^45^** in the CDR1, Thr**^57^** and Thr**^60^** in the C’ ß strand, Phe**^67^** and Lys**^69^** in the CDR2, and Thr**^110^** and Asp**^111^** in the CDR3. The FcµR-contacting aa residues of the IgM Cµ4 domain included Asn**^465^**, Leu**^466^**, Arg**^467^**, Glu**^468^**, and Glu**^526^** (Figure 6). Thus, none of the aa residues of FcµR predicted by our site-directed mutagenesis analysis were found to directly interact with IgM, but two of them, Asn**^66^** and Asn**^109^**, were immediately adjacent to the crystallographically identified contact residues Phe**^67^** and Thr**^110^**, respectively. Since, except for Thr**^57^**, six of the seven contact residues identified by crystallography are conserved in both human and mouse species, these residues were not selected by our site-directed mutagenesis strategy. The Lys**^79^**-Arg**^83^** stretch in the DE loop of FcµR was apparently not part of the IgM-binding interface, and hence, the finding of gain of constitutive IgM-binding by the mouse FcµR triple mutant remains puzzling and could be caused by an indirect conformational effect. Two Ig-like domains of FcµR were found to interact with both sides of the Cµ4 domain of the IgM monomer, suggesting a 2:1 stoichiometry for the FcµR/IgM monomer. Notably, both sides of the FcµR-binding Cµ4 domain of IgM BCR seemed to be accessible on the surface of B cells. In contrast, four FcµR molecules could bind the same side of the Cµ4 domain of an IgM pentamer in a way that once the first FcµR molecule had bound, then the remaining three receptors bound only to the same side in a certain cooperative manner, probably with the help of FcµR stalk regions, resulting in a 4:1 stoichiometry for the FcµR/IgM pentamer. The potential fifth Cµ4 domain was blocked by the J chain loop. According to the Rosenthal group, FcµR bound to both sides of the Cµ4 domain of the IgM pentamer (8:1 stoichiometry). The first FcµR binding was the site where pIgR D1 (or the secretory component) bound, probably due to its higher affinity. This suggested that FcµR could bind secretory IgM as well.

Collectively, crystallographic and cryo-EM analyses reveal intricate mechanisms for the interaction of FcµR with monomeric and pentameric IgM molecules. The identification of aa residues of FcµR critical for IgM-ligand binding would facilitate screening of individuals with inborn errors of immunity, particularly FcµR deficiency. 

## 5. Ex Vivo Functions of Human FcµR and Its Association with Diseases

### 5.1. Ex Vivo Functions

*FCMR* deficiency has not yet been identified in humans but, if it exists, the clinical abnormalities might be much more complex and profound than in *Fcmr*-ablated mice, because additional cell types (i.e., T and NK cells) express the human FcµR. Thus, functional analyses of human FcµR rely on ex vivo experiments and several findings are noteworthy. (i) Co-ligation of FcµR and other receptors (e.g., Fas, CD2, BCR) on the same cell surface by agonistic IgM mAbs either inhibits Fas-mediated apoptosis or enhances BCR- or CD2-mediated Ca^2+^ mobilization, suggesting a dual (negative or positive) signaling ability [7,34,41,42]. The *cis* engagement dominates the *trans*, probably due to locally high concentrations of IgM ligands [34]. (ii) FcµR is highly expressed by chronic lymphocytic leukemia (CLL) B cells [7,33,43,44,45] and, after IgM binding, it is rapidly internalized in lysosomes via an endocytic pathway [33]. FcµR is not only present on the plasma membrane, but is also accumulated in large pools in the *trans*-Golgi network [33]. (iii) FcµR on T and NK cells is dramatically down-modulated upon IL-2 stimulation in vitro, consistent with the finding that the surface levels of FcµR on freshly prepared effector memory T cells are much lower than on naïve T cells. Binding of IgM to FcµR on NK cells initiates intracellular signals but does not mediate NK cell cytotoxicity [42]. (iv) FcµR-mediated IgM uptake by T cells enhances their surface expression of the T cell receptor and co-stimulatory molecules, thereby facilitating T cell activation, particularly when peptide antigen concentrations are low [46]. 

### 5.2. Enhanced Levels of the Soluble Form of FcµR in Patients with Chronic Lymphocytic Leukemia

**Figure 7:**
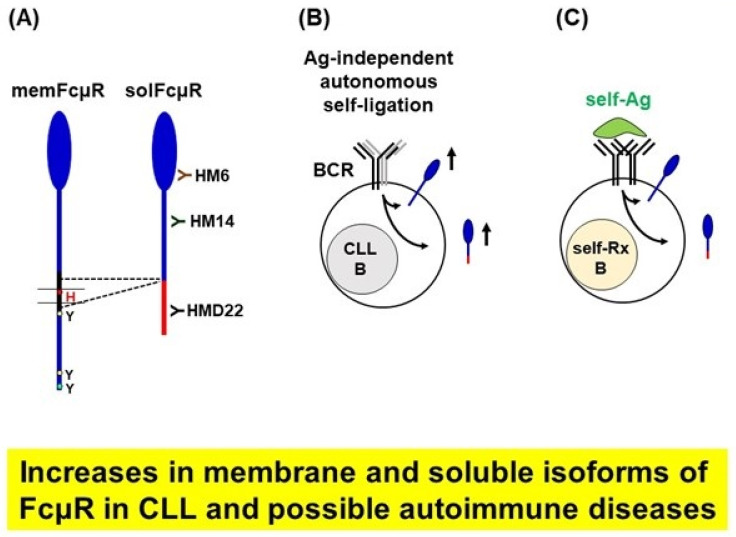
Enhanced expression of both membrane-bound and soluble isoforms of FcµR in patients with CLL and possible autoimmune diseases. (**A**) The novel 70-aa C-terminal hydrophilic tail of soluble FcµR (solFcµR), which results from alternative splicing of membrane FcµR (memFcµR) with skipping of the transmembrane exon, is highlighted in red. Three representative mAbs reactive with different epitopes on the solFcµR are shown. (**B**) Antigen-independent, autonomous self-ligation of BCR is the hallmark of the neoplastic CLL B cells. This may account for the enhanced expression of both memFcµR and solFcµR. (**C**) The hypothesis here is that persistent BCR stimulation with self-antigens, such as in antibody-mediated autoimmune diseases, results in increases of both memFcµR and solFcµR.s.

A variety of FcµR transcripts have been reported in the NCBI databases of both human and mouse species. However, only one splice variant other than its full length has been characterized at the protein level. It was originally identified in a phorbol myristate acetate-activated human pre-B cell line 697 by RT-PCR analysis with a set of primers corresponding to the translation initiation and termination sites of the human FcµR cDNA (NCBI gene accession: HM480394). It turned out that the alternatively spliced *FCMR* transcript resulted from the direct splicing of exon 4 (stalk-2) to exon 6 (cytoplasm-1), thereby skipping exon 5 (TM). This splicing event resulted in a frameshift in exon 6 and generated a novel 70-aa hydrophilic C-terminal tail, suggesting a soluble form of FcµR (solFcµR) (Figure 7A). The existence of such solFcµR in biological samples was confirmed when we assessed serum levels of FcµR in patients with CLL [45].

The association of the FcµR with CLL has long been suggested based on: (i) the ability of CLL cells to form rosettes with IgM-coated erythrocytes [47,48], (ii) IgM binding to CLL cells, as monitored by flow cytometry [49,50], and (iii) the expression of elevated levels of *FCMR* transcripts by microarray and RT-PCR analyses [43,44]. We also examined the surface expression of FcµR on the B and T cells obtained from CLL patients using receptor-specific mAbs and flow cytometry [7,45] (Figure 7B). CLL B cells (CD5**^+^**/CD19**^+^**) expressed much higher surface levels of FcµR than B cells from healthy donors. Such enhanced expression was more evident in better outcome or indolent CLL (i.e., Ig heavy-chain variable region (*IGHV*)-mutated, CD38**^−^**, or early Rai-stage) than in poor outcome or aggressive CLL (i.e., *IGHV*-unmutated, CD38**^+^**, or advanced Rai-stage). Notably, surface FcµR levels were also significantly elevated in non-CLL B cells (CD5**^−^**/CD19**^+^**) and T cells (CD19**^−^**/CD5**^+^**), especially in better outcome CLL patients, when compared with the corresponding populations in healthy donors [45]. 

To determine whether CLL patients contain any soluble or extracellular (e.g., shed) form of FcµR in their sera, we performed a sandwich enzyme-linked immunosorbent assay (ELISA) using one anti-FcµR mAb (HM6 clone) as a capture antibody for serum FcµR and a biotin-labeled anti-FcµR mAb (HM14 clone) as a detection reagent (both HM6 and HM14 mAbs recognize a different epitope in the EC region of FcµR). Many CLL patients clearly exhibited elevated serum titers of FcµR, albeit with a wide range, as compared with healthy donors, except for one (see below). The titers were strongly correlated with the number of blood lymphocytes but not with CLL outcome indicators, including *IGHV* mutational statuses and Rai stages. Serum FcµR was resolved as a protein with an *M*_r_ of ~40 kDa, thus being smaller than its membrane-bound counterpart of ~60 kDa (Figure 7B). By liquid chromatography tandem mass spectrometry analysis, the ~40 kDa serum FcµR was unequivocally defined as the aforementioned solFcµR. Notably, one of the tryptic peptides corresponded to the junction formed by splicing exon 4 to exon 6, ruling out the proteolytic cleavage of membrane-bound FcµR [45]. The cellular source of the solFcµR in CLL patients appeared to be both CLL B and non-CLL B cells, but not T cells, as determined by RT-PCR and ELISA of their cell populations isolated by the fluorescence-activating cell sorter.

Collectively, using receptor-specific mAbs, enhanced levels of both the ~60 kDa membrane-bound FcµR and the ~40 kDa solFcµR were demonstrated in patients with CLL compared with healthy donors. Enhanced surface FcµR expression was more evident in indolent CLL than aggressive CLL. Notably, the surface FcµR levels in both CD5**^-^** non-CLL B cell and T cell populations were also significantly increased, especially in indolent CLL. The serum levels of solFcµR in CLL patients strongly correlated with their circulating lymphocyte counts but not with outcome indicators. The solFcµR was a product of an alternatively spliced FcµR transcript by both CD5**^+^** CLL B and CD5**^-^** non-CLL B cells. 

### 5.3. Lessons from Studies on FcµR in CLL

Among various leukemias and lymphomas, the enhanced expression of FcµR transcripts or proteins is predominantly observed in CLL [51]. Unlike other B cell malignancies, BCRs on CLL cells are unique in that they can ligate each other via Ig heavy-chain CDR3 of one BCR with the framework region 2 of another BCR, regardless of their *IGHV* mutation statuses, thereby providing antigen-independent, cell-autonomous signaling [2,52] (Figure 7B). This antigen-independent homotypic BCR interaction on B CLL cells seems to affect the clinical course of disease as stronger affinity and longer half-life contacts are observed in indolent CLL, while weaker and more short-lived contacts are seen in aggressive CLL [53]. This unique property of CLL B cells could account for the selective enhancement of FcµR expression in CLL, consistent with the finding that cross-linkage of BCR on normal blood B cells with antibodies enhanced the surface expression of FcµR [7]. However, it was still unclear why surface FcµR levels were also elevated on non-CLL B and T cells, especially in indolent CLL patients. In this regard, CLL has been characterized by alterations of both adaptive and innate immune systems, including the roles of T cells, nurse-like cells, DCs, and bone marrow stromal cells in tumor surveillance and pathogenesis [54]. 

Another indication of the linkage between BCR ligation and enhanced FcµR expression was our observation of elevated levels of serum FcµR in one apparently ‘healthy’ individual who, however, developed high titers of anti-nuclear antibodies approximately two years after blood donation. We thus hypothesize that the production of solFcµR is a consequence of persistent BCR signaling. It is anticipated that the solFcµR levels may be elevated in individuals with other diseases characterized by chronic BCR stimulation, such as antibody-mediated autoimmune disorders (Figure 7C). SolFcµR may modulate B cell function, either as a decoy receptor or by interacting with IgM BCR. In this regard, it is noteworthy that administration of a recombinant solFcµR variant (human FcµR EC/IgG Fc fusion protein lacking complement binding) into experimental allergic encephalomyelitis (EAE)-susceptible mice ameliorates the myelin oligodendrocyte glycoprotein-induced EAE [17].

### 5.4. Generation of mAbs Specific for solFcµR

Even though there are no reports describing the shedding of membrane FcµR by proteolytic cleavage, mAbs specifically recognizing the unique C-terminal portion of solFcµR as capturing reagents would be ideal in sandwich ELISA for assessments of the solFcµR in sera from patients with CLL or autoimmunity. This capturing strategy is clearly favorable over the FcµR EC-specific mAb (see above; Figure 7A). To develop such solFcµR-specific mAbs, we initially employed Ag8 plasmacytoma stably transduced by native solFcµR cDNA. However, because of its poor production, we modified the native solFcµR cDNA construct by introducing a consensus Kozak sequence directly 5′ of the translation initiation site and inserting an 18 nucleotides coding six His residues tag before the termination codon. The resultant His-tagged solFcµR transductant produced 10-fold more solFcµR than the native solFcµR transductant. To our surprise, unlike the native solFcµR, the His-tagged solFcµR protein associated with extracellular membrane vesicles or exosomes, as well as with plasma membranes, rather than being in a free, secreted form [55]. Notably, the culture supernatants of His-tagged solFcµR transductants (even containing 10% fetal calf serum proteins) could be used for directly coating ELISA plates to immobilize solFcµR. This might imply that the exosome-attached form of His-tagged solFcµR proteins can efficiently adhere to polystyrene surfaces, as opposed to the free form of non-His-tagged solFcµR proteins. This unique property of His-tagged solFcµR was very convenient in the initial screening strategy by ELISA for hybridomas secreting solFcµR-specific mAbs without having to purify solFcµR proteins. In addition to this advantage, we also employed an in vivo differential immunization strategy using the solFcµR-His transductant as an immunogen and the membrane FcµR transductant as a tolerogen, resulting in generation of a new mouse IgG1κ mAb specific for human solFcµR [55]. This mAb (HMD22 clone) will be used for assessments of a large cohort of patients with CLL or autoimmune disorders to verify the above hypothesis of solFcµR being a disease indicator as well as to identify the cells secreting the solFcµR. 

## 6. Epilogue

Considering that IgM is the first phylogenetically emerging Ig isotype from jawed vertebrate onward, it seemed plausible to assume that *FCMR* orthologues are broadly distributed in various vertebrate species, as complement is. However, FcµRs appear to be selectively found in mammals [56]. Another unexpected finding is that the expression of FcµR is restricted to lymphocytes: B, T, and NK cells in humans, and only B cells in mice. This implies that the lymphocyte-specific FcµR possesses distinct effector functions as compared with the FcRs for switched Ig isotypes that are expressed by various cell types, including phagocytes. From studies of *Fcmr*-deficient mice, FcµR has a regulatory function in B cell tolerance, as evidenced by their propensity to produce autoantibodies of both IgM and IgG isotypes (see the review in [13]). In this article, we have introduced our comments or frank opinions about two recent articles describing conflicting views—the regulation of anti-tumor activity by FcµR-bearing phagocytes infiltrating around tumors [19] and the reverse transcytosis of secretory IgM by FcµR expressed on the apical surface of M cells in Peyer’s patches [27]. The signal function of the Ig-tail tyrosine (ITT)-like motif seen in the FcµR cytoplasmic tail is now formally demonstrated by substitution of the IgG2a ITT. Unresolved aspects associated with our previous experiments of FcµR, i.e., its dual-signaling ability and its fast migration on SDS-PAGE after receptor ligation, were reiterated, with small progress. Recent crystallographic structural analyses of human FcµR by two different groups of investigators reveal seven key aa residues of FcµR directly contacting with the fourth constant domain of IgM as well as intricate mechanisms for the interaction of FcµR with the IgM monomer and pentamer. Differences in such aa residues as compared with our previous findings based on site-directed mutagenesis were discussed. The functional significance of the soluble form of FcµR, an alternative splice variant skipping the TM exon, has just begun to be explored in diseases characterized by persistent BCR stimulation, such as CLL and antibody-mediated autoimmune disorders. If this article ultimately facilitates research activity in the FcµR field by new investigators to resolve the many puzzles and unresolved issues as well as the functions, especially of human FcµR, the authors will be grateful and satisfied. We hope that this article will open new avenues of investigation.
